# The Fabrication, Drug Loading, and Release Behavior of Porous Mannitol

**DOI:** 10.3390/molecules29030715

**Published:** 2024-02-04

**Authors:** Zhe Li, Xiaosui Luo, Qiong Li, Zhengji Jin, Abid Naeem, Weifeng Zhu, Lihua Chen, Yi Feng, Liangshan Ming

**Affiliations:** 1 Key Laboratory of Modern Preparation of TCM of Ministry of Education, Institute for Advanced Study, Jiangxi University of Chinese Medicine, Nanchang 330004, China; 20191004@jxutcm.edu.cn (Z.L.); luoxiaosui@jxutcm.edu.cn (X.L.); 20142046@jxutcm.edu.cn (Q.L.); 20211044@jxutcm.edu.cn (Z.J.); 20214003@jxutcm.edu.cn (A.N.); 19930220@jxutcm.edu.cn (W.Z.); 19940081@jxutcm.edu.cn (L.C.); fyi@vip.sina.com (Y.F.); 2 Engineering Research Center of Modern Preparation Technology of TCM of Ministry of Education, Shanghai University of Traditional Chinese Medicine, Shanghai 201203, China

**Keywords:** porous mannitol, PVP K30, co-spray–antisolvent, mechanism, adsorption kinetics, dissolution behavior

## Abstract

Porous materials are widely used as an effective strategy for the solubilization of insoluble drugs. In order to improve the solubility and bioavailability of low water-solubility drugs, it is necessary to prepare porous materials. Mannitol is one of the most popular excipients in food and drug formulations. In this study, porous mannitol was investigated as a drug carrier for low water solubility drugs. Its fabrication, drug loading, and drug release mechanisms were investigated. Porous mannitol was fabricated using the co-spray–antisolvent process and utilizing polyvinylpyrrolidone K30 (PVP K30) as the template agent. Porous mannitol particles were prepared by changing the proportion of the template agent, spraying the particles with mannitol, and eluting with ethanol in order to regulate their pore structure. In subsequent studies, porous mannitol morphology and characteristics were determined systematically. Furthermore, curcumin and ibuprofen, two poorly water-soluble drugs, were loaded into porous mannitol, and their release profiles were analyzed. The results of the study indicated that porous mannitol can be prepared using PVP K30 as a template and that the amount of template agent can be adjusted in order to control the structure of the porous mannitol. When the template agent was added in amounts of 1%, 3%, and 5%, the mannitol pore size increased by 167.80%, 95.16%, and 163.98%, respectively, compared to raw mannitol. Molecular docking revealed that mannitol and drugs are adsorbents and adhere to each other by force interaction. The cumulative dissolution of curcumin and ibuprofen-loaded porous mannitol reached 69% and 70%, respectively. The release mechanism of curcumin and ibuprofen from drug-loaded mannitol was suitable for the Korsmeyer–Peppas kinetic model. In summary, the co-spray–antisolvent method proved effective in fabricating porous materials rapidly, and porous mannitol had a remarkable effect on drug solubilization. The results obtained are conducive to the development of porous materials.

## 1. Introduction

In general, the oral administration of drugs is the most popular method of drug delivery since it is convenient, painless, associated with a higher level of patient compliance, and low-risk in terms of cross-infection [1]. Unfortunately, most therapeutic drugs have a limited oral bioavailability, likely due to their poorly soluble nature, low dissolution rates, low permeability, and inferior stability, all of which pose serious challenges to their therapeutic effectiveness [2,3]. Orally administered BCS class II or IV drugs are susceptible to poor aqueous solubility and consequently have poor dissolution rate (e.g., ibuprofen, danazol, carbamazepine, indomethacin, curcumin, griseofulvin, etc.) [4,5]. According to statistics, approximately no less than 40% of newly developed drugs are poorly water soluble, leading to a number of drug control issues, such as insufficient dosages [6]. Thus, improving the solubility of poorly water-soluble drugs may be beneficial to the pharmaceutical industry in order to meet the needs of users and meet product specifications [3,7,8].

The development of highly effective drug formulations for enhancing the solubility and/or dissolution rate of poorly soluble drugs and improving their oral bioavailability has been explored using a variety of approaches, which has proved to be more effective than developing new drugs. Existing studies have shown that dispersing drugs within porous materials can be an effective strategy for the solubilization of insoluble drugs. Several studies have demonstrated that porous materials can enhance the dissolution behavior of insoluble drugs [9,10]. Porous materials have a large specific surface area, high porosity, uniform pore distribution, and clear surface characteristics, which are conducive to improving the rate of drug release and dissolution [11]. Several methods can be used to fabricate porous materials, including the use of their own matrices, chemical synthesis, pore-forming agents, and template agents [12]. Among the four methods used to prepare porous particles, the first three require complex preparation techniques, whereas the porous carrier material prepared using its own matrix pore-forming method has a low level of drug loading. In contrast, porous materials prepared through chemical synthesis and the use of pore-forming agents leave behind an organic solvent residue. On the other hand, the preparation of porous materials using template agents is a relatively simple process, and no additional procedures are required. Most pore-forming agents currently available are volatile and easily decomposable. During particle preparation, pores are formed when pore-forming agents are removed from the solid matrix using high-temperature gas generation or sublimation [12]. Common pore-forming agents include ammonium bicarbonate (NH_4_HCO_3_), sodium bicarbonate (NaHCO_3_), camphor, menthol, and thymol [13,14,15,16], of which camphor, menthol, and thymol are insoluble in water, and their pore-forming principle is that they sublimate slowly to produce pores [17]; it is difficult for them to be completely removed. Furthermore, these three pore-forming agents are pharmacologically active, which will further cause problems related to drug safety and effectiveness. Conversely, sodium bicarbonate and ammonium bicarbonate undergo a chemical reaction that releases gases from the pores as they decompose [18]. On the one hand, this method generally produces residual ammonia and sodium ions, which are pollutants to the environment, while on the other hand, excessive sodium ions are harmful to the human body. Template agents are available in a variety of types, and the porous materials produced by the template agents contain no residue of harmful substances, and they can be completely removed afterwards. Co-spray-drying of the assistant template agent is the most common method to produce porous materials, and the common template agents include sucrose, citric acid, D-fructose, D-anhydrous glucose, etc. [19,20]. In this study, polyvinylpyrrolidone K30 (PVP K30) was used as a template agent for the preparation of porous materials. Moreover, PVP K30 can reduce the surface tension of the solution and the wall adhesion phenomenon during spray-drying, thus improving the yield.

There are a number of porous materials available on the market today, including porous silica, porous starch, porous lactose, etc. [21,22,23]. The porous structure could promote the effective delivery of drugs [24]. Some studies have reported that a combination of porous silica and solid dispersion technology was highly effective at achieving high drug solubilization [25,26]. Planinsek et al. [27] showed that the solubilization mechanism of porous materials involves superfine dispersion of drugs on a large surface area. In addition, drugs exist in an amorphous state and possess a high degree of energy. Ali et al. [28] developed a method for preparing porous starch and used porous starch to study carbamazepine solubility. The results showed that compared with pure carbamazepine, porous starch loaded with carbamazepine could completely release the drug in 20 min. Saffari et al. [29] showed that acetaminophen loaded in porous lactose could release 85% of the drug within 7 min, and its dissolution behavior was significantly improved compared with pure acetaminophen. Due to their high porosity and large surface area, porous materials are effective carriers to significantly improve the rate and behavior of drug dissolution. In addition to the above three common porous materials, a series of porous materials such as porous chitosan, porous calcium carbonate, and porous microspheres are widely used [30,31,32].

Recently, mannitol has become increasingly popular as an alternative to lactose in food and pharmaceutical formulations, and mannitol is widely used as a medicinal excipient due to its obvious advantages, including excellent taste, low hygroscopic property, good compactability, and chemical inertness to active ingredients in drugs [33]. It is an ideal additive and carrier for moisture-sensitive ingredients. However, the low porosity and specific surface area of mannitol limit its application as a drug carrier [34,35]. It has been observed that mannitol has poor dissolution properties when used as a carrier [36]. Dissolution behavior is an important characteristic of drugs for clinical efficacy. Therefore, when mannitol is used as a drug carrier, it is necessary to fabricate porous mannitol in order to improve the loading capacity of mannitol and the dissolution behavior of insoluble drugs. In published studies on porous mannitol, it was found that porous mannitol can be prepared by different methods and used as a carrier to effectively improve the dissolution of insoluble drugs. However, few studies have been conducted on the fabrication of porous mannitol using PVP K30 as a template agent. Studies on porous mannitol generally fail to consider the mechanisms of fabrication, the loading of drugs, and the mechanism of drug release.

**Figure 1:**
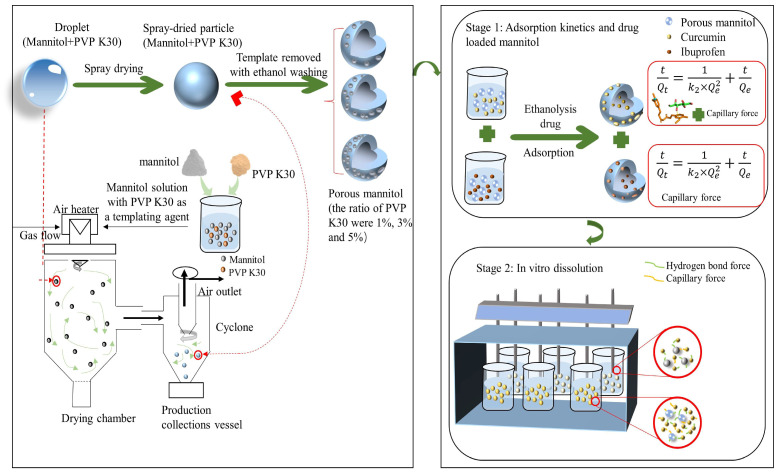
Schematic illustrations of preparation and application of porous mannitol.

Therefore, in this study, we employed the co-spray–antisolvent method with PVP K30 as a template to prepare porous mannitol, which was then utilized as a carrier for insoluble drugs such as curcumin and ibuprofen. Additionally, the mechanisms of fabrication, drug loading, and release behavior based on the porous mannitol were explored systematically. Schematic illustrations of preparation and application of porous mannitol are shown in Figure 1. First, we used PVP K30 as the template agent and combined the characteristics of PVP K30 dissolved in water and ethanol with co-spray–antisolvent methods, resulting in a co-spray complex between PVP K30 and mannitol. PVP K30 was then removed by ethanol elution since mannitol is not soluble in ethanol, whereas PVP K30 is readily soluble in ethanol. Mannitol with a porous structure was prepared. The fabrication mechanism of porous structure during the preparation of porous mannitol was discussed by changing the ratio of PVP K30. Subsequently, the structure and physical characteristics of the porous materials were characterized by various techniques. The adsorption behavior between drug and carrier was studied by adsorption kinetics. Molecular docking was used to study the interaction of mannitol with curcumin and ibuprofen, and the mechanism of drug loading and release using porous mannitol as carrier was discussed. This study provides novel perspectives, theoretical support, and practical guidance for the development of porous materials that are capable of being applied to a wide range of applications in the near future.

## 2. Results and Discussion

### 2.1. Characterization of the Material Properties

#### 2.1.1. Porous Mannitol before Drug Loading

The yield and MC of porous mannitol are shown in Table 1. After spray–drying, the yield of samples was high. The yield of Mannitol-P0 and Mannitol-P0’ were both 85.36%. The other yields were 82.19% for Mannitol-P1, 72.36% for Mannitol-P3, and 88.47% for Mannitol-P5. Compared with Mannitol-P0’, the yields of Mannitol-P1 (treated with 1% PVP K30) and Mannitol-P3 (treated with 3% PVP K30) decreased by 3.71% and 15.23%, respectively. In contrast, compared with Mannitol-P0’, the yield of porous Mannitol-P5 (treated with 5% PVP K30) increased by 3.64%. Studies have shown that PVP K30 has a high glass transition temperature (Tg) (174 °C) [37]. With the increased concentration of PVP K30 as the matrix-forming agent, the Tg of the whole powder sample was increased to overcome the sticky wall problem in the drying process. Therefore, theoretically, the yield of sample powder was proportional to the amount of PVP K30. However, the Tg of mannitol was noticeably low (11 °C) [38], which would be the main reason for the irregular change of powder yield. In addition, the size of the spray droplets during the spray-drying process can also affect the overall yield. The surface tension of the spray solution determines the size of the spray droplets. Higher surface tension leads to larger droplets, whereas lower surface tension results in smaller droplets. When larger droplets are formed during spray-drying, they are more likely to adhere to the dryer wall before complete drying. This adherence to the wall hampers the drying process and reduces the overall spray-drying yield [39,40]. The MC of all samples ranged from 0.12% to 0.58% with low humidity content.

The fabrication of porous mannitol includes two processes: co-spray-drying, which is the fabrication of composite particles containing template agent, and ethanol washing, which is the removal of the template agent. In this study, PVP K30 was used as the template agent and composite particles containing template agent were prepared by co-spray-drying with mannitol. The template agent contained in the composite particles was removed due to PVP’s properties of being easily soluble in water and ethanol, while mannitol is insoluble in ethanol, and the porous mannitol was prepared [41]. Moreover, the porous structure can be regulated according to the amount of PVP.

**Figure 2:**
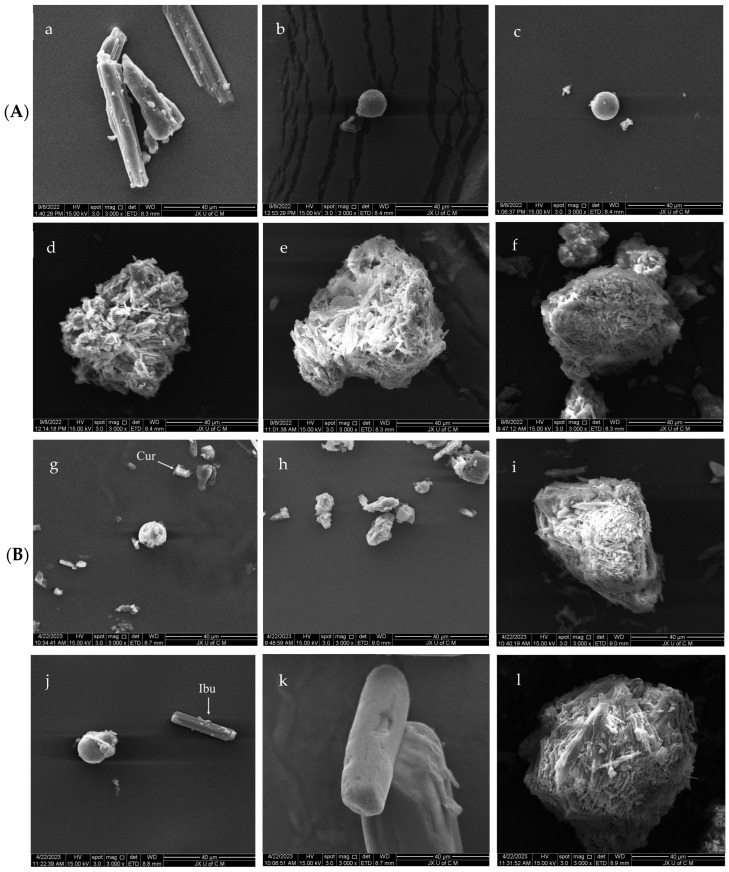
SEM analysis. (**A**) SEM images of mannitol before drug loading; (**B**) SEM of mannitol after drug loading. (**a**) Mannitol, the raw material; (**b**) Mannitol-P0, prepared without template agent PVP and ethanol; (**c**) Mannitol-P0’, processed without PVP but with ethanol; (**d**) Mannitol-P1, processed with 1% PVP; (**e**) Mannitol-P3, processed with 3% PVP; (**f**) Mannitol-P5, processed with 5% PVP; (**g**) Mannitol-P0’-Cur, the Mannitol-P0’ loaded with curcumin; (**h**) Cur, the model drug of curcumin; (**i**) Mannitol-P3-Cur, the Mannitol-P3 loaded with curcumin; (**j**) Mannitol-P0’-Ibu, the Mannitol-P0’ loaded with ibuprofen; (**k**) Ibu, the model drug of ibuprofen; (**l**) Mannitol-P3-Ibu, the Mannitol-P3 loaded with ibuprofen.

In order to verify the successful preparation of porous mannitol, SEM was conducted on the samples at different magnifications (500× and 3000×). The surface morphology of the single particle sample in the powder is shown in Figure 2A (3000×), and the surface morphology of the powder samples is shown in the Supplementary Materials (Figure S1). The powder sample was composed of more single particles, and the surface morphology of single particles in the powder sample showed that the surface morphology of spray-dried mannitol (Figure 2b–f) was significantly different from that of raw mannitol (Figure 2a). When Mannitol-P0 and Mannitol-P0’ were compared, the mannitol samples processed without PVP K30 (Figure 2b,c) showed that the mannitol samples obtained by spray-drying were spherical or quasi-spherical, and there was no difference in morphology between them, indicating that the pure ethanol elution process would not destroy the structural properties of mannitol. Porous mannitol (Figure 2d–f) exhibited a fluffy porous structure similar to that of sea urchins. With the increase of the proportion of PVP K30, the surface of the porous mannitol displayed fluffier and more compact pores, with improved sphericity. Due to the presence of PVP K30 in the preparation process of porous mannitol, the surface of these porous particles presents a structure different from that of Mannitol-P0 and Mannitol-P0’. The results of SEM showed that the porous mannitol was prepared successfully by the co-spray–antisolvent method.

To further verify the surface properties of porous mannitol, the SA experiment was performed, and the results of SA-BET, SA-BJH, pore volume (PV), and pore diameter (PD) are shown in Table 1. All these results quantificationally verified the successful preparation of porous mannitol. Compared with raw mannitol, the SA-BET of Mannitol-P1, Mannitol-P3, and Mannitol-P5 increased by 231.8%, 443.8% and 198.3%, respectively; the SA-BJH of these samples increased by 114.4%, 285.4% and 15.8%, respectively. Mannitol-P1, Mannitol-P3, and Mannitol-P5 exhibited 4.7-fold, 6.5-fold, and 1.7-fold higher PV than raw mannitol, respectively. Similarly, they also showed 1.6-fold, 0.9-fold, and 1.6-fold higher PD than raw mannitol, respectively. Compared with Mannitol-P0’, the SA-BET values of Mannitol-P1, Mannitol-P3, and Mannitol-P5 increased by 69.4%, 177.6% and 52.2%, respectively; the SA-BJH values of Mannitol-P1, Mannitol-P3, and Mannitol-P5 increased by 86.2%, 234.8%, and 0.5%, respectively. However, for PV, the values of Mannitol-P1 and Mannitol-P3 increased by 84.3% and 141.4% when compared with Mannitol-P0’, respectively. Similarly, for PD, the values of Mannitol-P1 and Mannitol-P5 were increased by 12.8% and 11.2% when compared with those of Mannitol-P0’, respectively.

Interestingly, when the proportion of PVP K30 increased (Table 1), the SA, PV, and PD of porous mannitol did not increase proportionally. A possible explanation for this discrepancy would be the fact that the spray-drying process involves complex mass and heat transfer. In addition, the change in atomization pressure, outlet temperature, and the difference of dispersion degree of PVP K30 in the spray-drying process might also cause the disproportionate change. Compared with Mannitol-P0 and Mannitol-P0’ (Table 1), porous mannitol showed the same changes in SA-BET and SA-BJH. In porous mannitol, the SA of 5% PVP K30 (Mannitol-P5) porous mannitol demonstrated a decreasing trend, which might be due to the limited pore-forming ability of the template agent, which further confirmed that excessive template agent could not increase the SA of porous particles proportionally. Therefore, the SA of the sample did not necessarily increase with the increase of the PVP K30 ratio. Furthermore, the SA, pore diameter and pore volume of the sample did not increase proportionally with the ratio of PVP K30, which might be due to the pore number changing with the increase of the addition of PVP K30. The disproportionate increase in SA can also lead to irregular changes in pore volume and pore diameter. These findings were consistent with Jiang’s research on the influence of PVP dosage and molecular weight on porous film coatings that enhance the air permeability of fabrics [42].

It was also found that the values of *ρ_b_*, *ρ_t_*, and *ρ_true_* of porous mannitol were low and decreased by 35.48~37.60%, 16.80~25.52%, and 1.92~2.35% compared with the raw mannitol, respectively (Table 1). The lower density values indicated that the porous mannitol had a loose structure, which again indicated that the porous mannitol was successfully prepared by the co-spray–antisolvent method, and the obtained porous mannitol had better filling and drug loading properties.

The particle size of the prepared mannitol particles was characterized, and the results are shown in Table 1. Interestingly, porous mannitol, as compared to raw mannitol and Mannitol-P0, exhibited a smaller particle size and a more uniform particle size distribution (Table 1). In general, span and uniformity were inversely related to the particle size distribution of materials [43,44]. Compared with raw mannitol, the span of Mannitol-P1, Mannitol-P3, and Mannitol-P5 increased by 65.1%, 43.6%, and 47.7%, respectively; the uniformity of Mannitol-P1, Mannitol-P3, and Mannitol-P5 increased by 69.9%, 47.3% and 54.7%, respectively; however, the d (0.5) of Mannitol-P1, Mannitol-P3, and Mannitol-P5 decreased by 43.1%, 32.4%, and 38.9% respectively. However, compared with Mannitol-P0’, the span of Mannitol-P1, Mannitol-P3, and Mannitol-P5 decreased by 23.5%, 33.4%, and 31.5%, respectively; the uniformity of Mannitol-P1, Mannitol-P3, and Mannitol-P5 decreased by 22.3%, 32.7%, and 29.2%, respectively; unlike Mannitol-P1, the d (0.5) of Mannitol-P3 and Mannitol-P5 increased by 16.0% and 4.8%, respectively. The results indicated that span, uniformity, and d (0.5) of porous mannitol did not change proportionately with an increased PVP K30 ratio. Briefly, when compared with raw mannitol and Mannitol-P0’, the span, uniformity, and d (0.5) of porous mannitol were significantly different. The reasons for this phenomenon might be as follows: the processing method of the material determined the particle size of the material. The shape and particle size of particles were also affected by the outlet temperature. Different outlet temperatures lead to different particle shapes and size [45]. In addition, adding PVP K30 to the mannitol solution can not only reduce the surface tension of the sample solution, but also make the particles show smaller and more uniform size distribution after spray-drying. The results showed that the lower surface tension would lead to smaller droplets of the spray, thus reducing the particle size of the sample [40]. With the increase of the proportion of PVP K30, the surface tension of the sample solution did not increase proportionately, which might be another important reason for the irregular change of the particle size of porous mannitol (Table 2). Kawakamia et al. [46] showed that droplet size and particle size after spray-drying had a good correlation with surface tension on the millisecond time scale, regardless of the type of surfactant. The smaller the surface tension, the smaller the particle size after spray-drying. Zhu et al. [41] found that in the preparation of porous lactose, PVP K30 not only significantly reduced the surface tension of the sample solution, but also produced smaller and more uniform particles, which was similar to the results of our study.

The flowability of powder is very important to the design of production process. It is usually characterized by angle of repose (AR), CI, and HR [41,47]. In general, the flowability of the sample is inversely proportional to the above three parameters; that is, the smaller the parameter value, the better the flowability [43]. Compared with Mannitol-P0’, the values of CI and HR of porous mannitol decreased by 10.0~21.8% and 7.6~15.2%, respectively. In comparison to raw mannitol, the AR, CI, and HR of non-ethanol mannitol (Mannitol-P0) and porous mannitol prepared by spray-drying increased by 17.08~22.24%, 28.19~73.79%, and 11.08~24.74%, respectively. Table 1 shows that porous mannitol generated greater AR, CI, and HR values than the raw mannitol, indicating poor flowability. The particle size results also confirmed the conclusion that porous mannitol had greater span and uniformity. This might be because the porous mannitol structure prepared with PVP K30 as the template is not smooth, causing the formation of mechanical chimeric particles, resulting in poor flowability of the powder. Secondly, the particle size obtained by the Buchi B-290 laboratory-grade spray-dryer is generally within the range of 1~10 μm [48], and the powder has strong electrostatic and van der Waals forces in the particle size stage, resulting in poor overall flowability of the powder.

**Figure 3:**
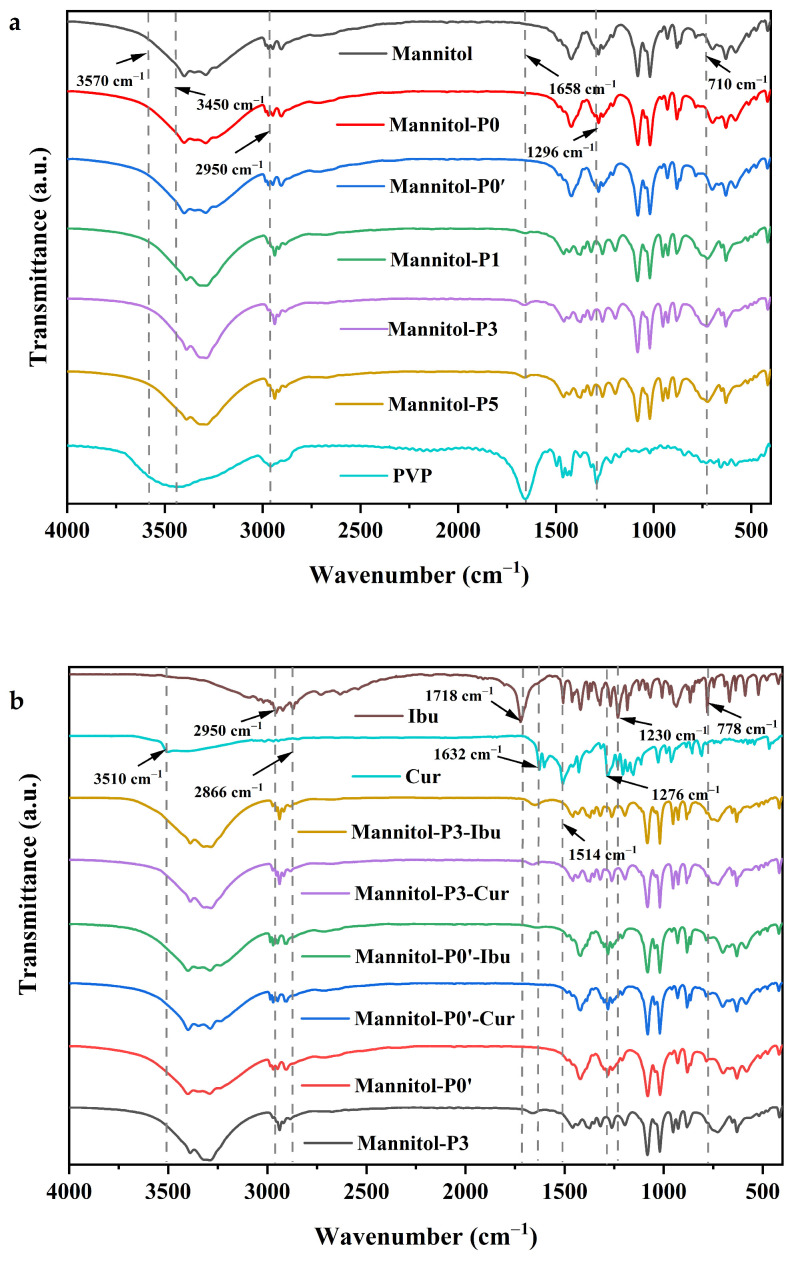
FTIR spectra. (**a**) FTIR spectra of samples after different treatments; (**b**) FTIR spectra of drug-loaded mannitol. Mannitol, the raw material; Mannitol-P0, prepared without template agent PVP and ethanol; Mannitol-P0’, processed without PVP but with ethanol; Mannitol-P1, processed with 1% PVP; Mannitol-P3, processed with 3% PVP; Mannitol-P5, processed with 5% PVP; PVP, polyvinylpyrrolidone K30; Ibu, the model drug of ibuprofen; Cur, the model drug of curcumin; Mannitol-P3-Ibu, the Mannitol-P3 loaded with ibuprofen; Mannitol-P3-Cur, the Mannitol-P3 loaded with curcumin; Mannitol-P0’-Ibu, the Mannitol-P0’ loaded with ibuprofen; Mannitol-P0’-Cur, the Mannitol-P0’ loaded with curcumin.

The chemical composition of mannitol samples was analyzed with FTIR. The results of FTIR analysis (Figure 3a) confirmed that the prepared porous mannitol particles were mostly similar and had slight differences from raw mannitol, Mannitol-P0, and Mannitol-P0’. In addition, Mannitol-P0 and Mannitol-P0’ had similar absorption peaks on the infrared spectrum, which indicated that the chemical composition of mannitol was not affected by ethanol elution. The FTIR analysis revealed that the infrared absorption peaks of porous mannitol did not significantly change with the increase in PVP K30 ratio. Characteristic peaks of raw mannitol, Mannitol-P0, Mannitol-P0’ and porous mannitol could be found nearby, at 3570~2950 cm^−1^ and 1658~710 cm^−1^, respectively (Figure 3a). Specifically, the sample that was all porous mannitol had a clear peak at 710 cm^−1^. The single peak of raw mannitol and non-porous mannitol changed into several small peaks that were not obvious in the wavelength range of 1658~1296 cm^−1^. Another finding was that the tensile vibration of the carbonyl group was most obvious in the PVP K30 infrared spectrum, which usually appears around 1658 cm^−1^; a broad peak displayed at 3450 cm^−1^ wavelength of the sample of PVP in FTIR, which was shown as the O-H tensile vibration of absorbing water [49], but the characteristic peak did not exist in the infrared spectrum of porous mannitol prepared using PVP K30 as the template, which proved that PVP K30 in porous mannitol had been eluted cleanly.

The raw mannitol, Mannitol-P0, and Mannitol-P0’ showed very similar stretching vibration spectra of C-H and O-H between 3570 cm^−1^ and 2950 cm^−1^ and O-H deformation spectra between 1658 cm^−1^ and 710 cm^−1^ [50]. Secondly, for raw mannitol, there was a clear absorption peak at 3450 cm^−1^ due to the O-H-free vibration of water molecules [41]. In addition, the FTIR spectra also significantly showed the bands of PVP K30 at 2950 cm^−1^, 1658 cm^−1^, and 1296 cm^−1^, representing the tensile vibration of C-H, C=O, and O-H, respectively. All the porous mannitol in the infrared spectra had the same peak at the wavelength of 710 cm^−1^, and the single peak of raw mannitol with the scope of 1658~1296 cm^−1^ changed into multiple peaks in porous mannitol, which might have been due to the chemical shift of mannitol caused by the addition of PVP K30 during spray-drying because the O-H of mannitol reacted with C=O in PVP K30, resulting in the change of structure.

**Figure 4:**
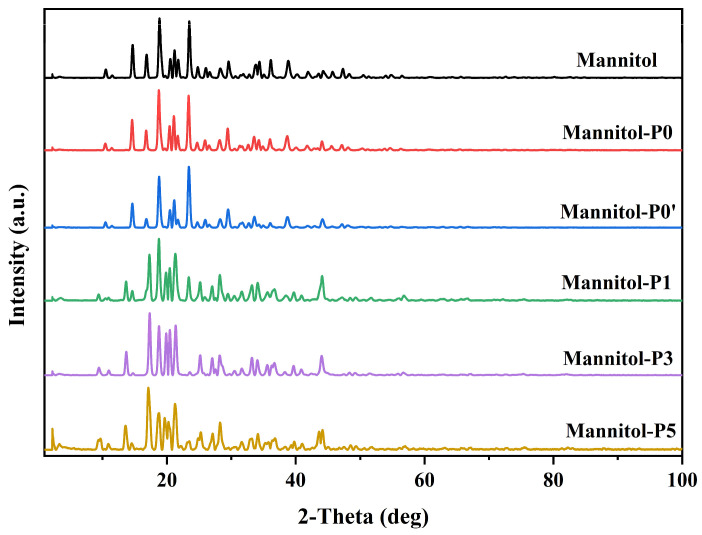
X-ray diffractograms of samples. Mannitol, the raw material; Mannitol-P0, prepared without template agent PVP and ethanol; Mannitol-P0’, processed without PVP but with ethanol; Mannitol-P1, processed with 1% PVP; Mannitol-P3, processed with 3% PVP; Mannitol-P5, processed with 5% PVP; PVP, polyvinylpyrrolidone K30.

XRD is the fastest and most direct method to determine the basic structure information of crystalline materials. The XRD results of the samples powder revealed that the crystal morphology of mannitol changed when PVP K30 was added during the preparation of samples (Figure 4). The curves showed that the main crystallization peaks of mannitol at 2θ had values of approximately 9.5°, 14°, 16°, 19°, and 23°. Smith et al. [51] investigated the influence of the polycrystalline type on the surface energy of the mannitol crystal type. In their study, it was shown that α-polycrystalline mannitol was characterized by peaks at 2θ values of 9.5°, 13.8°, and 17.4°, which further supports the results of this study. Furthermore, it can be clearly seen from Figure 4 that there was basically no difference between Mannitol-P0 and Mannitol-P0’, indicating that whether ethanol was used or not in the Mannitol-P0 and Mannitol-P0’ had no substantial effect on the spray-dried particles. Compared with raw mannitol, the peak shape of porous mannitol was significantly changed, and significant differences could be observed around 23° and 46°. After PVP K30 was added, the peak intensity of the sample weakened to around 23° and was divided into several small peaks. Porous mannitol has a sharp peak near 2θ = 46°, which is significantly different from raw mannitol, Mannitol-P0, and Mannitol-P0’, which might be due to the fact that PVP K30, as the most effective crystallization inhibitor, has an inhibitory effect on the crystal shape and thus changes the peak shape [52].

**Figure 5:**
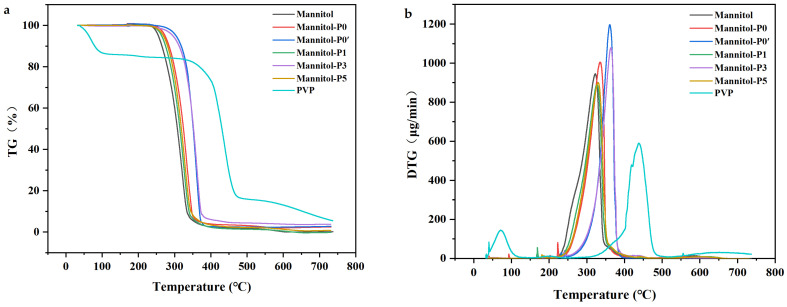
TG and DTG thermograms of samples. (**a**) TG thermograms; (**b**) DTG thermograms. Mannitol, the raw material; Mannitol-P0, prepared without template agent PVP and ethanol; Mannitol-P0’, processed without PVP but with ethanol; Mannitol-P1, processed with 1% PVP; Mannitol-P3, processed with 3% PVP; Mannitol-P5, processed with 5% PVP; PVP, polyvinylpyrrolidone K30.

Decomposition, evaporation, and desorption events are accompanied by mass changes within the sample [53]. The decomposition behaviors and the thermal stability of samples were studied by thermogravimetric analysis (TGA) under isothermal conditions. Typical thermal degradation curves of TGA are shown in Figure 5. The TG curve showed that the sample’s decomposition can be divided into three parts [54]. The first part (0~180 °C) was mainly the low-temperature stage, in which the free water or surface-attached water in the sample was generally evaporated. The second part (180~500 °C) was the evaporation of volatile substances and weightlessness. During this stage, except for PVP K30, all the other samples showed rapid weightlessness and finally reached mass equilibrium. The third part (500~800 °C) was the passive zone, where coke was formed [55]. With the increasing temperature, the peak value of all samples reached a maximum value between 300 °C and 400 °C. It could also be clearly observed from the curves that raw mannitol, Mannitol-P0, Mannitol-P0’, and porous mannitol all followed a one-step weight loss rule at 0~700 °C. The weightlessness of mannitol was obvious within the range of 210~300 °C; the weight loss rate was faster, before 275 °C; and the mass balance was reached at 350 °C. The weight loss of Mannitol-P0, Mannitol-P0’, and porous mannitol was obvious within the range of 270~310 °C; the weight loss rate was faster, before 300 °C; and the mass balance was reached at 360 °C.

It could also be clearly observed that the stability of porous mannitol varies with the ratio of PVP K30 (Figure 5). Firstly, the overall weightlessness level of porous mannitol was smaller than that of Mannitol-P0’, which might be because the porous mannitol was in a crystallized state, which was a relatively stable state [41]. XRD results also confirmed the presence of a crystalline form in porous mannitol. Secondly, from the derivative thermogravimetric (DTG) curves (Figure 5b), Mannitol-P1 and Mannitol-P5 showed a similar weightlessness state, the curves were basically identical, and the peak DTG temperature was 340 °C. The peak temperature of porous mannitol with 3% PVP K30 was 370 °C, indicating an increase of 30 °C. As fine powders were used during analysis, heat transfer resistance had a negligible effect [54]. This temperature difference was attributed to the greater stability of porous mannitol prepared with the addition of 3% PVP K30 compared to the other two ratios. In addition, the peak temperatures of Mannitol-P0 and Mannitol-P0’ were also higher than that of raw mannitol, indicating that the stability of mannitol after spray-drying was higher than that of raw mannitol, and the stability of the sample was improved [56].

In summary, porous mannitol was successfully prepared with the co-spray–antisolvent method. Then the materials’ properties were characterized systematically. The results showed that the prepared porous mannitol has large specific surface area and porosity, and its surface was like a fluffy sea urchin.

#### 2.1.2. Porous Mannitol after Drug Loading

The surface morphology of the single particle in the powder samples (Figure 2B) and powder samples (Figure S1) after loading was also characterized in order to further understand the changes in powder properties after drug loading. From the surface morphology results of the single particle sample after drug loading (Figure 2B), when Mannitol-P0’ (Figure 2c) was used as a carrier, the surface morphology of drug-loaded Mannitol-P0’ changed (Figure 2g,j); a part of the drug was adsorbed into the carrier, and the other part was still scattered in the form of the free drug, indicating that the drug could not be completely adsorbed when Mannitol-P0’ was used as a drug carrier. Upon successful adsorption, the drug and the carrier would exist as inclusion complexes. Similarly, when Mannitol-P3 was used as a carrier, the surface morphology of Mannitol-P3 loaded with curcumin (Figure 2i) and ibuprofen (Figure 2l) were significantly different from that of unloaded Mannitol-P3 (Figure 2e), and the surface structure of Mannitol-P3 loaded with drugs was more compact. However, the surface of unloaded Mannitol-P3 was fluffy and porous, like a sea urchin. The strong contrast between the two (Figure 2e vs. Figure 2i, Figure 2e vs. Figure 2l) indicated that the drug could be successfully loaded into the carrier when Mannitol-P3 was used as a carrier. The surface morphology of powder samples also proved this conclusion. When Mannitol-P0’ was used as the carrier, a part of the drug was adsorbed to the carrier, while the other part of drug still existed in its free form (Figure S1). When Mannitol-P3 was used as a carrier, the drug was successfully adsorbed onto the carrier (Figure S1).

In order to clarify the possible interaction between the drug and mannitol, the samples loaded with the drug were characterized by FTIR (Figure 3b). The model drug of curcumin exhibited an obvious absorption peak at 3510 cm^−1^, which corresponded to the O-H stretching vibration of phenolic groups in curcumin. The other two distinct absorption peaks of curcumin were located at the wavelengths of 1514 cm^−1^ and 1276 cm^−1^, respectively. There was a clear absorption peak at 1632 cm^−1^ due to the presence of carbonyl groups in curcumin [57]. For the curcumin-loaded Mannitol-P3 and Mannitol-P0’, the characteristic peaks corresponding to curcumin were not found in the infrared spectrum. The absence of an absorption peak at 1632 cm^−1^ indicated that the carbonyl group in curcumin may form hydrogen bonds with the hydroxyl group in the carrier, and force interaction occurs between the two. In addition, the infrared curves of Mannitol-P3 and Mannitol-P0’ did not change significantly before or after drug loading. The results showed that when Mannitol-P3 and Mannitol-P0’ were used as carriers, they effectively adsorbed the model drug curcumin, indicating that curcumin was successfully loaded into the sample.

For ibuprofen, the absorption peak in the wavelength range of 3120~2950 cm^−1^ could be attributed to the stretching vibration of aromatic C-H in the chemical structure. The absorption peak in the range of 2950~2866 cm^−1^ could be attributed to the stretching vibration of aliphatic C-H in the chemical structure. The sharp absorption peaks of ibuprofen at 1718 cm^−1^ and 1230 cm^−1^ were due to the tensile vibration of the carboxyl group and the tensile vibration of the C-C bond, respectively. Another obvious absorption peak of ibuprofen was located at the wavelength of 778 cm^−1^, which was represented by the vibration of CH_2_ [58]. Similarly, for Mannitol-P3 and Mannitol-P0’ loaded with ibuprofen, no characteristic peak corresponding to ibuprofen was found in the infrared spectrum, which might be due to the ibuprofen forming internal inclusions with the carrier, indicating that ibuprofen was successfully loaded into the sample.

**Figure 6:**
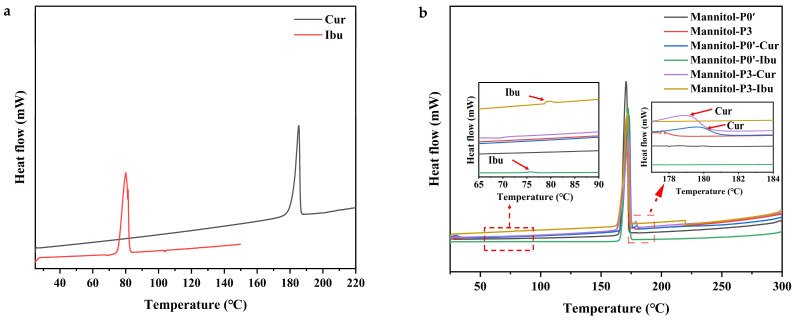
DSC thermograms. (**a**) DSC thermogram of curcumin and ibuprofen; (**b**) DSC thermogram of drug-loaded samples. Mannitol-P0’, processed without PVP but with ethanol; Mannitol-P3, processed with 3% PVP; Mannitol-P0’-Cur, the Mannitol-P0’ loaded with curcumin; Mannitol-P0’-Ibu, the Mannitol-P0’ loaded with ibuprofen; Mannitol-P3-Cur, the Mannitol-P3 loaded with curcumin; Mannitol-P3-Ibu, the Mannitol-P3 loaded with ibuprofen.

DSC has been widely used to determine the physical states of active components in samples. The DSC results for samples are shown in Figure 6. We could clearly observe that melting peaks appeared in all samples based on the DSC curves, indicating that the prepared samples were crystalline. This was consistent with the results determined by XRD. For the model drug curcumin, its melting peak was found to occur at 183 °C (Figure 6a). Compared with Mannitol-P0’, curcumin-loaded Mannitol-P0’ showed a small peak near 180 °C, which might be the melting peak of curcumin. However, the position of the peak deviated from that of the pure drug. This deviation can be attributed to the interactions between curcumin and mannitol. Similarly, compared with Mannitol-P3, the melting peak of Mannitol-P3 loaded with curcumin was located at 179 °C, which was also offset from that of pure drug, providing further evidence of potential interactions between curcumin and mannitol. This conclusion was consistent with the infrared results of drug-loaded powder discussed above.

The melting peak of the model drug ibuprofen was located at 80 °C (Figure 6a). Similarly, compared with Mannitol-P0’, Mannitol-P0’ loaded with ibuprofen showed a small peak at 76 °C, indicating the melting peak of ibuprofen. Compared with Mannitol-P3, a small peak of Mannitol-P3 loaded with ibuprofen appeared near 78 °C, representing the melting peak of ibuprofen, but the positions of the two peaks were shifted from that of pure drug, which might be due to the force between ibuprofen and mannitol. Interestingly, the melting peak of the pure drug was obvious, while that of powder was small, which might be due to the higher concentration of pure drug and the smaller carrier adsorption capacity of powder. The melting peak of Mannitol-P3 loaded with ibuprofen was more obvious than that of Mannitol-P0’ loaded with ibuprofen, indicating that the adsorption capacity of Mannitol-P3 loaded with ibuprofen was larger than that of Mannitol-P0’ loaded with ibuprofen. This further proved that the fabrication of porous materials was successful. The results of DSC showed that the melting peak of the drug-loaded powder was shifted due to the interaction between curcumin/ibuprofen and mannitol when compared with the melting of pure drug.

**Figure 7:**
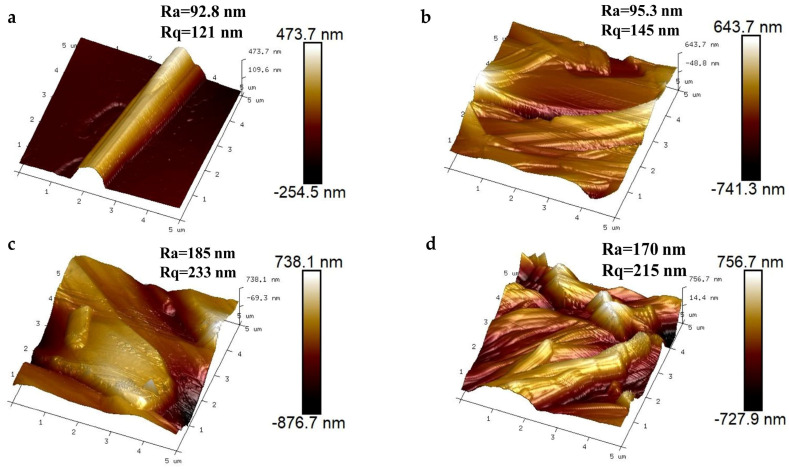
AFM images of drug-loaded porous mannitol. (**a**) Mannitol-P0’, processed without PVP but with ethanol; (**b**) Mannitol-P3, processed with 3% PVP; (**c**) Mannitol-P3-Cur, the Mannitol-P3 loaded with curcumin; (**d**) Mannitol-P3-Ibu, the Mannitol-P3 loaded with ibuprofen.

The Mannitol-P0’ and Mannitol-P3 before and after loading drugs were characterized by AFM (Figure 7). The mean roughness (Ra) and the mean square roughness (Rq) are commonly used to evaluate the surface roughness of a sample. The larger the value, the larger the surface roughness of the sample and the more complex the pore structure [59]. In AFM measurement, the light-colored area represents the relatively high area, while the dark area represents the relatively low area. It could be observed from the figure that the height difference of these samples was relatively obvious, indicating that the surface of the samples was complex and diverse. According to the AFM results, the Ra of Mannitol-P0’ was 92.8 nm, and the Rq was 121 nm. The sample surface height was relatively regular. The surface morphology and roughness of Mannitol-P3 were different before and after drug loading. Before drug loading, the surface height of Mannitol-P3 was relatively irregular, and the surface was shallow convex, indicating pores. The value of Ra was 95.3 nm, and the value of Rq was 145 nm. Both of the two parameters of Mannitol-P3 were larger than those of Mannitol-P0’, indicating that the surface roughness of Mannitol-P3 was much larger than that of Mannitol-P0’, and the pore structure of Mannitol-P3 was complex. After drug loading, the size of Mannitol-P3 varied with the drug loading amount. The Ra of Mannitol-P3 loaded with curcumin was 185 nm and the Rq was 233 nm, and the surface roughness was relatively high. The surface morphology of Mannitol-P3 loaded with ibuprofen was similar to that of Mannitol-P3 loaded with curcumin; the Ra and the Rq were 170 nm and 215 nm, respectively. The values of both parameters were smaller than those of Mannitol-P3 loaded with curcumin, indicating that the surface roughness of Mannitol-P3 loaded with curcumin was higher than that of Mannitol-P3 loaded with ibuprofen. The results indicated that curcumin-loaded mannitol had a more complex pore structure, which made the binding force between the drug and the carrier weak. AFM results showed that porous mannitol was successfully prepared by the co-spray–antisolvent method, and there was interaction between the curcumin/ibuprofen and mannitol. These results were also consistent with DSC characterization.

### 2.2. Molecular Docking Analysis

**Figure 8:**
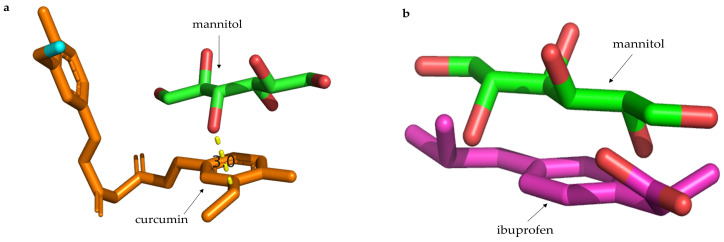
Docking results of model drugs and mannitol. (**a**) Optimal conformation of curcumin and mannitol. (**b**) Optimal conformation of ibuprofen and mannitol.

Molecular docking is a method to study intermolecular interactions at the molecular level and to predict intermolecular binding patterns and affinity [60]. The molecular docking results of the drug and mannitol showed that there was a hydrogen bond force between the curcumin and mannitol (Figure 8a). In addition to the hydrogen bond force, capillary force was another major force between the curcumin and mannitol [61]. The combination of the two forces made the binding between curcumin and mannitol more stable, but the binding force was weak. However, the molecular docking results of ibuprofen and mannitol showed that there was no force in the form of a hydrogen bond between them, but the two molecules were close to each other (Figure 8b), indicating a tendency of interaction between molecules. In this case, the dominant force contributing to the interaction between ibuprofen and mannitol was the capillary force. The FTIR results of the drug-loaded powder confirmed the interaction between the model drug and mannitol. In addition, the DSC analysis of drug-loaded powder found that the position of the drug peak in drug-loaded powder was shifted from that of pure drug, which further confirmed the existence of force interactions between the drug and mannitol.

### 2.3. Adsorption Kinetics and In Vitro Dissolution Behavior

#### 2.3.1. Adsorption Kinetics Analysis

**Figure 9:**
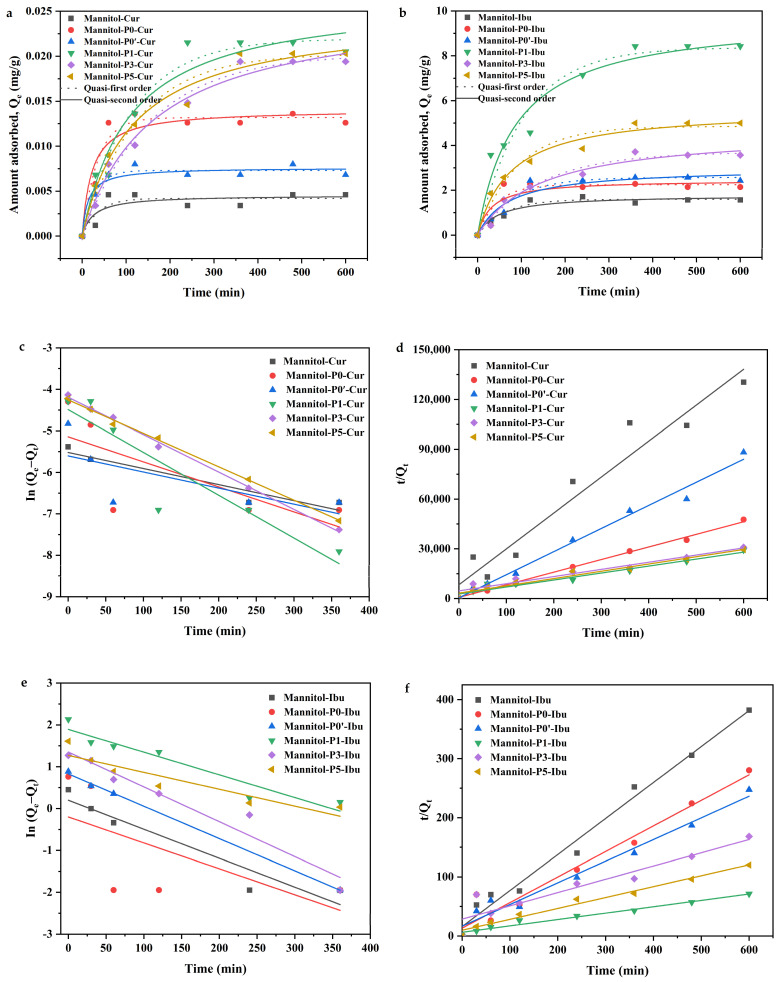
Adsorption kinetics curves of the materials. (**a**) Adsorption kinetics curve of model drug curcumin. (**b**) Adsorption kinetics curve of model drug ibuprofen. (**c**) The fitting curves of the quasi-first order adsorption kinetics of curcumin. (**d**) The fitting curves of the quasi-second order adsorption kinetics of curcumin. (**e**) The fitting curves of the quasi-first order adsorption kinetics of ibuprofen. (**f**) The fitting curves of the quasi-second order adsorption kinetics of ibuprofen. Mannitol, the raw material; Mannitol-P0, prepared without template agent PVP and ethanol; Mannitol-P0’, processed without PVP but with ethanol; Mannitol-P1, processed with 1% PVP; Mannitol-P3, processed with 3% PVP; Mannitol-P5, processed with 5% PVP.

The model drugs, curcumin and ibuprofen, displayed adsorption capacities different from the porous mannitol material. In this study, a linear correlation was observed between the concentration of curcumin and its absorbance in the range of 1.05~6.30 μg/mL. The regression equation was A = 0.1762 C − 0.0279, R^2^ = 0.9991 (*n* = 6). Similarly, a linear correlation was found between the concentration of ibuprofen and its absorbance in the range of 100.3~702.1 μg/mL. The regression equation was as follows: A = 0.0014 C + 0.0326, R^2^ = 0.9997 (*n* = 6). The porous mannitol was loaded with curcumin and ibuprofen, and the adsorption kinetics were investigated, which are shown in Figure 9. For the two model drugs, when the adsorption time was gradually extended and the adsorption amount of the drug also increased from the adsorption kinetic curve of the material, it finally reached the stable state of adsorption. The adsorption capacity of each sample tended to be stable when the adsorption time reached 6 h. In addition, it can be clearly observed from the adsorption kinetics curves of the samples that for curcumin, the amount of adsorption of Mannitol-P0 was significantly higher than that of mannitol powder and Mannitol-P0’. The amount of adsorption of Mannitol-P1, Mannitol-P3, and Mannitol-P5 in porous mannitol was slightly different. For the model drug ibuprofen, the amount of adsorption of Mannitol-P0 was smaller than that of Mannitol-P0’, and the amount of adsorption of three porous mannitol was significantly different. This indicated that the selection of different model drugs would cause obvious differences in the amount of adsorption when the drug adsorption vector was the same. The results showed that the adsorption capacity of curcumin and ibuprofen as model drugs were 0.02 mg/g and 8 mg/g (Figure 9a,b), respectively. One possible reason is that there was a force interaction between the drug and mannitol, but the force between the curcumin and mannitol was significantly weaker than that between the ibuprofen and mannitol, and the weaker force made most of the drug molecules free and difficult to bind to the adsorbent or carrier [62], which was confirmed by the results of AFM and molecular docking. In addition, another reason might be that the differences in the size and arrangement of drug molecules and the size and shape of porous mannitol particles could lead to differences in the dispersion of drug molecules in porous mannitol pores [63].

Adsorption kinetics can be generally divided into two categories; one is the adsorption reaction model, and the other is the adsorption diffusion model. The process of drug adsorption and diffusion on the adsorbent can be achieved by the following three steps; that is, the first is the diffusion of the drug on the liquid film near the adsorbent, followed by the diffusion into the pores of porous structure containing liquid—this process is also called intra-particle diffusion—and finally the adsorption and desorption between the drug and the adsorbent [64]. In this study, the quasi-first order and the quasi-second order adsorption kinetics model were fitted to the adsorption curve. The fitting results are shown in Table 3 and the fitting curves are shown in Figure 9c–f. In the particle diffusion kinetics model, the fitting curve of In (*Q_e_* − *Q_t_*) to *t* was a straight line that did not pass through the origin, indicating that the adsorption rate of the adsorbent was affected by both intraparticle diffusion and liquid film diffusion. This shows that the four fitting curves were all correlated (Table 3), indicating that the two fitting models can effectively fit the adsorption results and capture all the adsorption processes. These results were consistent with the kinetic results of particle diffusion. In addition, according to the correlation coefficient R^2^ for the two model drugs, the values of the quasi-second order adsorption kinetics model were higher than those of the quasi-first order adsorption kinetics model (Table 3), indicating that the quasi-second order adsorption model was more suitable for the process of dynamic adsorption of porous mannitol and the two model drugs.

#### 2.3.2. Dissolution Analysis

**Figure 10:**
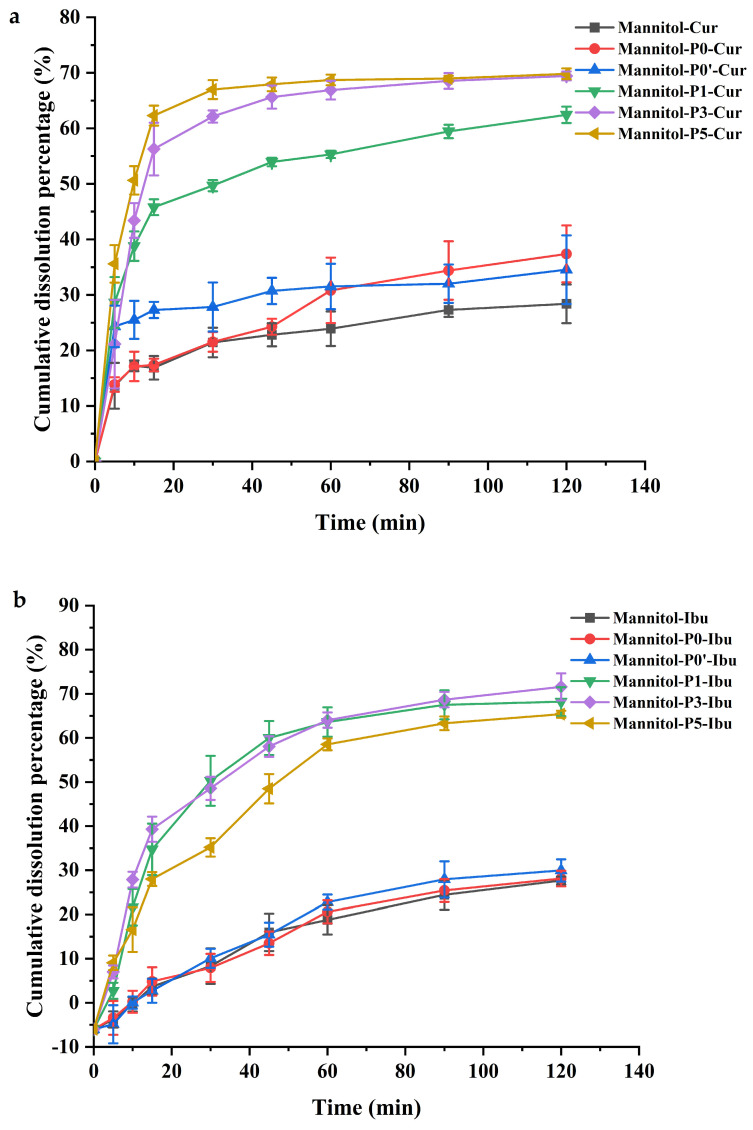
Dissolution profiles of drug-loaded porous mannitol. (**a**) In vitro dissolution profiles of curcumin-loaded mannitol. (**b**) In vitro dissolution profiles of ibuprofen-loaded mannitol. Mannitol, the raw material; Mannitol-P0, prepared without template agent PVP and ethanol; Mannitol-P0’, processed without PVP but with ethanol; Mannitol-P1, processed with 1% PVP; Mannitol--P3, processed with 3% PVP; Mannitol-P5, processed with 5% PVP.

The in vitro dissolution curves of mannitol particles loaded with curcumin and ibuprofen are shown in Figure 10, and the maximum cumulative release rate is shown in Table 4. Compared with porous mannitol, the solubilizing behavior of curcumin-loaded into Mannitol-P0 and Mannitol-P0’ was similar to that of curcumin-loaded into raw mannitol (Figure 10a). They all dissolved very slowly. Compared with raw mannitol, Mannitol-P0, and Mannitol-P0’, the dissolution behavior of porous mannitol loaded with curcumin improved significantly (Table 4). The dissolution rate of Mannitol-P3 and Mannitol-P5 was faster by between 5 min and 20 min. The dissolution equilibrium of Mannitol-P3 and Mannitol-P5 containing curcumin was basically reached at 45 min, and the dissolution equilibrium of Mannitol-P1 containing curcumin was also reached at 60 min. In addition, for porous mannitol loaded with curcumin, the final cumulative dissolution percentages of Mannitol-P1, P3, and P5 were 62%, 69%, and 69%, respectively. The dissolution behavior of porous mannitol loaded with curcumin was significantly faster than that of raw mannitol, Mannitol-P0, and Mannitol-P0’, which might be due to the higher SA and PV of porous mannitol.

Similarly, Mannitol-P0 and Mannitol-P0’ loaded with ibuprofen showed dissolution behavior similar to that of the raw mannitol (Figure 10b). The cumulative dissolution rate was low, and the dissolution behavior was poor. Compared with raw mannitol, Mannitol-P0, and Mannitol-P0’, the dissolution equilibria of Mannitol-P1, P3, and P5 containing ibuprofen were also reached at 60 min, and those of ibuprofen-loaded Mannitol-P1, P3, and P5 at 120 min were 68%, 70%, and 64%, respectively. Compared with raw mannitol and non-porous mannitol containing ibuprofen, the dissolution rate and dissolution behavior of the porous mannitol containing ibuprofen was significantly improved (Table 4). For ibuprofen-loaded porous mannitol (Mannitol-P1 and Mannitol-P5), the dissolution rate and cumulative dissolution percentage of Mannitol-P1 were significantly faster and higher than those of Mannitol-P5, which was contrary to the result of a curcumin-loaded model drug. The finding showed that PVP K30 ratio affected the improvement of drug dissolution behavior, and dissolution rate did not accelerate when PVP K30 ratio was increased. Similar to the dissolution behavior results of porous mannitol containing curcumin, the dissolution behavior of porous mannitol containing ibuprofen was significantly faster than that of the raw mannitol, Mannitol-P0, and Mannitol-P0’, which might be due to the higher SA and PV of porous mannitol. For ibuprofen-loaded raw mannitol, Mannitol-P0, and Mannitol-P0’, their dissolution behavior was the same as those of curcumin-loaded raw mannitol, Mannitol-P0 and Mannitol-P0’.

For the two model drugs, Mannitol-P3 had the highest cumulative dissolution percentage (Figure 10) and had the best effect on improving the dissolution behavior of insoluble drugs. This might be due to the values of SA and PV of Mannitol-P3, which were significantly higher than those of Mannitol-P1 and Mannitol-P5.

In addition, it can also be seen from the dissolution curve that the drug loaded within the porous mannitol dissolved rapidly, within the first 20 min, and changed with the addition of PVP K30. This might be because the surface of porous mannitol showed fluffier and denser pores after higher amounts of PVP K30 were added. This is further explained by the fact that the prepared porous mannitol had a large SA and large pores, and the SEM results also confirmed that the structure of the prepared porous mannitol was porous. In a series of characterization results of the drug-loaded powder, it was found that the dissolution behavior of the powder loaded with curcumin was significantly faster than that loaded with ibuprofen in the first 20 min. One possible reason is that the force interaction between curcumin/ibuprofen and mannitol significantly affected the dissolution behavior of the drug. This conclusion was consistent with the molecular docking results of drug-loaded powders discussed above.

Studies have shown that the loading of theophylline with porous silica as a carrier can change the dissolution time of theophylline by 50% within 14~405 min [65]. Similarly, when carvedilol (CAR) was loaded with mesoporous silica nanoparticles and mesoporous carbon nanoparticles, 70% of the drug was released rapidly within 90 min, and the dissolution behavior of CAR was improved [66]. Compared with other porous carriers, porous mannitol as a carrier can also achieve 70% drug release within 90 min; the dissolution behavior of insoluble drugs was improved effectively. The results showed that there is practical significance and potential application value to improving the solubility of insoluble drugs by using porous mannitol as carrier.

In order to further explore the drug release kinetics, three common mathematical models (the zero-order, first-order and Korsmeyer–Peppas kinetic models) were used to fit the in vitro release kinetics of drug-loaded mannitol. The results are shown in Table 5. It can be seen that the zero-order and first-order kinetic models were unsuitable for the model drug release from porous mannitol, while the Korsmeyer–Peppas kinetics model was the best model to explain the mechanism of drug release. Secondly, according to R^2^, the Korsmeyer–Peppas model of drug-loaded mannitol showed the highest R^2^ value compared with other mathematical models. Studies have shown that *n* in the Korsmeyer–Peppas model represents the drug release profile [67,68]. When *n* was less than 0.5, it referred to the drug diffusion release mechanism; when *n* was more than 1, it represented the drug erosion release mechanism; when *n* was more than 0.5 and less than 1, it indicated that the drug release was caused by the combination of diffusion and erosion mechanisms. In this study, the *n* value of all drug-loaded mannitol was less than 0.5, indicating that the drug release resulted from the diffusion mechanism. Therefore, the best model for releasing drugs from drug-loaded mannitol was the Korsmeyer–Peppas kinetic model.

The rate of solids’ dissolution in solution was usually positively correlated with SA [69,70], and the relationship between the two can be expressed using the Noyes–Whitney formula, given below:

(1) $$\frac{dC}{dt}=\frac{DS_{w}\left(C_{s}-C\right)}{Vh}$$

where *C* is the concentration of the dissolved substance at a given time *t*, *C_s_* is the solubility concentration of the substance, *D* is the diffusion coefficient of the substance, *S_w_* is the surface area of the exposed solid, *V* is the volume of the solvent, and *h* is the thickness of the diffusion layer [71,72].

Firstly, porous mannitol containing curcumin/ibuprofen exhibited superior dissolution behavior compared to raw mannitol, Mannitol-P0, and Mannitol-P0’ due to higher SA and PV (Table 1). Studies have shown that high SA and PV are due to hollow and porous structures due to uneven particle size distribution and significantly accelerate the dissolution of insoluble drugs [41]. In the particle size analysis of porous mannitol, we found that the particle size of the porous material increased disproportionately when the amount of PVP K30 was increased, and this pattern was also found in the flowability detection of porous mannitol, which indicated that the amount of PVP K30 has a strong influence on the behavior of porous mannitol as a carrier to improve the dissolution of insoluble drugs.

Secondly, the in vitro dissolution experiment further demonstrated that the release of the two model drugs were both consistent with the Korsmeyer–Peppas kinetic model. The cumulative dissolution percentage of insoluble drugs was the highest when the ratio of PVP K30 was 3%. As described in the SEM results, the porous mannitol surface showed fluffier and denser pores with the increase of PVP K30, and the morphology, structure, and porosity of the composite particles changed during the assembly and rearrangement.

Thirdly, studies have shown that the release of bioactive substances from the matrix occurs primarily as a result of the dissolution of the material’s surface, diffusion, and desorption [73]. In this study, quasi-first order and quasi-second order adsorption kinetics models were used to evaluate the adsorption curves to clarify the adsorption kinetics mechanism of the insoluble drugs curcumin and ibuprofen (Table 3). From the fitting accuracy of correlation coefficient R^2^, for the two model drugs curcumin and ibuprofen, their values in the quasi-second order adsorption kinetics model were higher than those in the quasi-first order adsorption kinetics model. Therefore, the quasi-second order adsorption kinetics model was more suitable to characterize the adsorption mechanism of insoluble drugs curcumin and ibuprofen. Additionally, in terms of adsorption capacity, the adsorption capacity of curcumin on the same carrier was significantly lower than that of ibuprofen, indicating that the force between curcumin and mannitol was weaker than that between ibuprofen and mannitol. Based on the results of the molecular docking experiment, mannitol and curcumin showed hydrogen bonding and capillary binding interactions. However, there was only capillary force between ibuprofen and mannitol.

## 3. Materials and Methods

### 3.1. Materials

PVP K30 was supplied by Ashland (Shanghai, China). Mannitol was obtained from Cofan Biotech Co., Ltd. (Guangzhou, China). Curcumin and ibuprofen were supplied by Maclean Bio-Tech Co., Ltd. (Shanghai, China). Spectral-grade potassium bromide was obtained from Aladdin Bio-Tech Co. (Shanghai, China). Further specific information about the compounds can be found in the Supplementary Material (Table S1).

### 3.2. Fabrication of Porous Mannitol

Several samples were prepared under the condition that the concentration of mannitol solution (10%, *w*/*w*) was fixed and that different amounts of template agents were used in aqueous solutions (1%, 3%, 5%) (*w*/*w*). They were labeled Mannitol-P1, Mannitol-P3, and Mannitol-P5, respectively. The samples were stirred at room temperature for 30 min to obtain a clear, transparent solution. Then, the samples were spray-dried using Buchi Mini Spray-dryer B-290 (BUCHI Co., Ltd., Flawil, Switzerland). The inlet temperature was 150 °C, the material pumping speed was 3~3.5 mL/min, and the atomization pressure was 45 bar. The spray-dried powder was collected and was mixed with ethanol at a ratio of 1:10 and stirred twice on a magnetic stirrer at 300 r/min for 12 h each time to eliminate the template agent. After the stirring, the porous sample was separated from the ethanol solution containing PVP K30 by centrifugation at 5000 r/min for 10 min, then dried in a vacuum drying oven at 40 °C for 12 h.

The yield (%) of dry powder was calculated by the following equation [41]:

(2) $$Yield=\frac{m_{1}}{m_{0}}\times 100$$

where *m*_1_ (g) was the quantity of the dried powder collected after vacuum drying oven treatment, *m*_0_ (g) was the total quantity of mannitol powder and PVP K30 in Mannitol-P0, Mannitol-P0’, Mannitol-P1, Mannitol-P3 and Mannitol-P5.

The raw mannitol properties were also investigated. The non-porous mannitol prepared without the template agent PVP K30 and ethanol treatment was labelled Mannitol-P0. The non-porous mannitol processed without PVP K30 but with ethanol was labelled Mannitol-P0’, and the process was the same for the preparation of porous mannitol.

### 3.3. Drug Loading Process

In this study, curcumin and ibuprofen were used as model drugs. Each drug was weighed (100.00 mg) and added to 30 mL of anhydrous ethanol to obtain an alcoholic solution of the drugs. Each solution was then mixed with 5.0 g of porous mannitol using a magnetic stirrer at 300 r/min for 7 h, centrifuged at 5000 r/min for 10 min, and then the supernatant was discarded. The drug-loaded mannitol was pre-dried with nitrogen, and then dried at 40 °C to a constant weight in a vacuum-drying oven. Then the drug-loaded powder was successfully obtained and used for the subsequent dissolution experiment.

### 3.4. Characterization of the Material Properties of Prepared Samples

#### 3.4.1. The Surface Tension of the Solution

The surface tension values of the different sample solutions were measured with a BZY-1 surface tensiometer (Shanghai Balance Instrument Factory, Shanghai, China) at normal pressure and room temperature (25 ± 1 °C). The platinum plate was rinsed and calibrated with distilled water before measurement. About 15 mL of the sample solution was subjected to measurement; the platinum plate immersion depth was 5 mm. Each measurement was repeated at least twice to determine reproducibility [46,74].

#### 3.4.2. Moisture Content, Particle Size, Flowability and Density

The moisture content (MC) of the sample was determined with a HE 53/02 Moisture Tester (Mettler Toledo Instrument Shanghai Co., Ltd., New York, NY, USA). The sample powder of 2.000 g was evenly spread on the sample plate and dried at 105 °C. Results were recorded when the MC changed less than 0.05% over a period of two consecutive 30 s [75].

The particle size of the material was analyzed using the Malvern 2000 laser diffractometer (Malvern Instruments Ltd., Malvern, UK). Measurements were taken on the instrument equipped with a Malvern 300 lens. The wet method was applied, and the solvent used in the wet method was anhydrous ethanol. The values of d (0.5), span, and uniformity were calculated by Mastersizer 2000 software.

The bulk density (*ρ_b_*) and tapped density (*ρ_t_*) of the sample were measured with a BT-1000 powder characteristics tester (Bettersize instrument Co., Ltd., Shanghai, China). The true density (*ρ_true_*) of the sample was measured with an AccuPyc II 1340 True Density Tester (Micromeritics Instruments Ltd., Shanghai, China). In addition, Carr’s index (CI) and Hausner’s ratio (HR) can be expressed with these two kinds of density, while the relationship between CI and HR with bulk density and trapped density can be expressed by the following equations.

(3) $$CI=\frac{\left(\rho_{t}-\rho_{b}\right)}{\rho_{t}}$$

(4) $$HR=\frac{\rho_{t}}{\rho_{b}}$$

#### 3.4.3. Atomic Force Microscopy (AFM)

A Bruker Dimension Icon atomic force microscope (Berlin, Germany) was used to measure the surface roughness and morphology of the samples before and after drug loading. Before measurement, part of the sample was dispersed into ethanol solution, then a few drops of the dispersed liquid were added to the mica sheet one by one, dried at room temperature of 25 °C, and scanned in the range of 5 μm × 5 μm. Finally, the surface morphologies of different samples were reconstructed in three dimensions.

#### 3.4.4. Surface Morphology

The apparent morphology of the samples was observed with a FEI Quanta 250 scanning electron microscope (Brno, Czech) (SEM) at an accelerating voltage of 15 kV. All samples were sputter-coated with gold to increase the conductivity, and then observed at magnifications rate of 500× and 3000× [76].

#### 3.4.5. Specific Surface Area (SA)

The nitrogen absorption isotherm of the samples was measured with a TriStar3000 specific surface analysis device (Micromeritics Instrument Corp., Norcross, GA, USA) in 77 K liquid nitrogen. The SA, pore volume (PV), and pore diameter (PD) were measured. The SA of samples could be measured with the Brunauer–Emmet–Teller (BET) method, and the pore volume of samples could be measured by the Barrett–Joyner–Halenda (BJH) method. The samples were treated with nitrogen for 4 h at 40 °C before formal determination [41].

#### 3.4.6. Fourier Transform Infrared Spectrometer (FTIR)

The samples were analyzed in the scanning range of 4000 cm^−1^~400 cm^−1^ and a resolution of 2 cm^−1^ by PerkinElmer FTIR spectrum 2 (Liantrisant, UK). Before testing, the samples were treated with potassium bromide at a ratio of 1:100, and then the powder mixtures were compressed into tablets at a pressure of 30 MPa. The background was scanned first before scanning the samples [77].

#### 3.4.7. X-ray Diffraction (XRD)

The crystallinity of the samples was determined by a XtaLAB PRO II diffractometer (Rigaku Co., Ltd., Tokyo, Japan). The crystal morphology of mannitol, PVP K30, Mannitol-P0, Mannitol-P0’, Mannitol-P1, Mannitol-P3, and Mannitol-P5 were studied in the scanning range of 1~100°. Radiation parameters for the Cu Kα radiation generated during this process were set to a current of 1 mA and a voltage of 50 kV.

#### 3.4.8. Thermogravimetric (TG) and Differential Scanning Calorimetry (DSC)

The samples were studied with EXSTAR TG/DTA6000 thermogravimetric detector (SII Nano, Kyoto, Japan), and the thermodynamic information of the samples was obtained. Briefly, about 5 mg of the sample was added to the pre-balanced ceramic pan, then the temperature was increased from 30 °C to 800 °C at a rate of 10 °C/min at a nitrogen flow rate of 200 mL/min [78].

The DSC studies of the samples were conducted using a PerkinElmer DSC 8000 differential scanning calorimeter (Shelton, CT, USA). During the experiment, a blank sealed cauldron was prepared first, and then samples of about 10 mg each were loaded into the sealed cauldron for detection according to the set procedure, in which each sample temperature was increased from room temperature of 25 °C to the detection temperature of different samples at the heating rate of 10 °C/min [79].

### 3.5. Molecular Docking

To investigate the greatest possible molecular conformation between mannitol and the model drug, molecular docking between the model drug and mannitol was performed using AutoDock Tools software (version 1.5.6). To begin with, the 3D structural data of drugs and mannitol were downloaded from the Pubchem database in SDF format. Then, OpenBabel software (version 2.3.2) was used to convert SDF format files downloaded from Pubchem into PDB format files. Finally, AutoDock Tools software was used to process acceptors and ligands [80].

### 3.6. Adsorption Kinetics and In Vitro Dissolution Experiment

#### 3.6.1. Adsorption Kinetics

Alcohol solutions of curcumin and ibuprofen were prepared according to the standard curves of the model drugs (curcumin and ibuprofen), and then 0.5 g of porous mannitol particles were added as adsorbent to the respective alcohol solutions of the model drugs, and the adsorption effects of adsorbents were observed at room temperature (25 °C) and stirred at a speed of 300 r/min. The samples were collected at 0, 30, 60, 120, 240, 360, 480, and 600 min, respectively, and filtered with a 0.45 μm filter. Curcumin and ibuprofen solutions were analyzed by UV-2550 UV-VIS (Shimadzu Joshi Co., Kyoto, Japan) spectrophotometer at wavelengths of 425 nm and 264 nm, respectively.

The adsorption amount of curcumin and ibuprofen (mg/g) was calculated using the following equation [81]:

(5) $$Q_{e}=\frac{\left(C_{0}-C_{e}\right)\times V}{m}$$

where *C*_0_ was expressed as the initial concentration of curcumin or ibuprofen, and *C_e_* was expressed as the equilibrium concentration of curcumin or ibuprofen, both in mg/L; *m* was expressed as the mass with porous particles as the carrier, and the unit was g; *V* represented the volume of curcumin or ibuprofen ethanol solution, the unit was mL; *Q_e_* (mg/g) represented the adsorption capacity of adsorbent with porous material as the carrier.

In addition, the adsorption kinetics were measured to determine the influence of time on the adsorption rates of various mannitol samples prepared in the study [82]. Adsorption kinetics can be expressed in a variety of models. Among them, based on solid adsorption capacity, Lagergren’s first-order rate equation, namely quasi-first order adsorption kinetic equation, was employed to determine liquid phase adsorption kinetics. Based on the assumption that the chemisorption mechanism can control the reaction rate of samples, a quasi-second order adsorption kinetics model was proposed, in which chemisorption was represented by the electron transition between adsorbent and adsorbent. The above two kinetic models can be calculated by following equations [83,84]:

(6) $$In\;\left(Q_{e}-Q_{t}\right)=InQ_{e}-k_{1}\times t$$

(7) $$\frac{t}{Q_{t}}=\frac{1}{k_{2}\times Q_{e}^{2}}+\frac{t}{Q_{e}}$$

where *Q_t_* was expressed as the adsorption capacity of the sample at any time *t* (min), and *Q_e_* was expressed as the equilibrium adsorption capacity of the sample, both in mg/g. *k*_1_ represented the rate constant of the quasi-first order model, and *k*_2_ represented the rate constant of the quasi-second order model.

#### 3.6.2. In Vitro Dissolution Experiment

A ZRS-8G intelligent dissolution instrument (Tianda Tianfa Technology Co., Ltd., Tianjin, China) was used to investigate the dissolution behavior of curcumin-containing mannitol and ibuprofen-containing mannitol. The slurry turning method was employed. Briefly, 200 mL of phosphate salt solution with a pH = 6.8, containing 5% Tween 80, was used as the dissolution medium. The temperature was kept constant at 37 ± 0.5 °C, and the paddle speed was set to 100 r/min. Sampling (5 mL) was performed at 5, 10, 15, 30, 45, 60, 90, and 120 min, and fresh solubilizing medium of the same volume was added immediately. The collected samples were filtered with a 0.45 μm filter and analyzed at 425 nm and 264 nm with a UV-2550 UV-VIS spectrometer (Shimadzu Joshi Co., Kyoto, Japan), respectively.

## 4. Conclusions

In this study, porous mannitol particles were successfully prepared by the co-spray–antisolvent method, which was used as a carrier for studying the dissolution behavior of poorly water-soluble drugs (curcumin and ibuprofen). Compared with other methods, the fabrication technology of porous mannitol with PVP as template is simple and does not require additional procedures. Moreover, due to the fact that PVP is easily soluble in water and ethanol, while mannitol is insoluble in ethanol, the template agent in the composite particles containing PVP prepared by co-spray-drying can be removed without residual harmful substances. The pore structure of porous mannitol can be regulated according to the amount of PVP. The study results showed that: (i) The porous mannitol prepared by the co-spray–antisolvent method had a larger SA and larger pores, and the SA-BET of porous mannitol increased by 231.8%, 443.8%, and 198.3% compared with the raw mannitol, respectively. The pore size was 1.6-fold, 0.9-fold, and 1.6-fold higher, respectively, than that of the raw mannitol; (ii) the behavior of porous mannitol can be regulated by PVP K30 concentration—the surface morphology and properties of porous mannitol vary with the addition of PVP K30 concentration; (iii) the molecular docking results showed that the drugs and mannitol were adsorbed and wrapped by force interaction. The adsorption kinetics of insoluble drugs were studied, and it was found that the quasi-second order adsorption kinetics model was more suitable to characterize the dynamic adsorption process of porous mannitol than quasi-first order adsorption kinetics model; (iv) porous mannitol was found to significantly improve the dissolution characteristics of curcumin and ibuprofen when used as a carrier, with a cumulative dissolution percentage of 69% and 70%, respectively, indicating a significant change in dissolution behavior; and the release mechanisms of the two model drugs were suitable for the Korsmeyer–Peppas kinetic model. Porous mannitol (Mannitol-P3) containing 3% PVP K30 was more suitable for drug loading and had the highest cumulative dissolution percentage for both drugs. This study demonstrated that porous materials can be prepared and regulated by the co-spray–antisolvent method, and the prepared porous materials can improve the dissolution behavior of insoluble drugs. The results can provide theoretical support and reference for the development of porous materials. In the future, we will conduct more researches on different porous materials comprehensively. For example, the effects of different fabrication technologies, technology parameters and prescription ratios on the structure of porous materials will be compared. We hope to explore the best preparation method for different porous materials, and promote the application of porous materials in different fields.

## Figures and Tables

**Table 1:** The characterization of basic properties of materials.

Materials	Yield (%)	MC (%)		*ρ_b_* (g/mL)		*ρ_t_* (g/mL)		*ρ_true_* (g/mL)		CI			HR	
Mannitol	/	0.17 ± 0.03		0.5053 ± 0.0046		0.6829 ± 0.0062		1.4966 ± 0.0017		26.0019 ± 0.0007			1.3514 ± 0.0000	
Mannitol-P0	85.36	0.12 ± 0.03		0.4513 ± 0.0099		0.6772 ± 0.0090		1.4785 ± 0.0015		33.3327 ± 2.3099			1.5012 ± 0.0531	
Mannitol-P0’	85.36	0.15 ± 0.00		0.3600 ± 0.0106		0.6567 ± 0.0067		1.4919 ± 0.0026		45.1875 ± 1.0514			1.8248 ± 0.0347	
Mannitol-P1	82.19	0.23 ± 0.03		0.3260 ± 0.0053		0.5495 ± 0.0114		1.4679 ± 0.0016		40.6660 ± 1.1534			1.6858 ± 0.0331	
Mannitol-P3	72.36	0.58 ± 0.03		0.3153 ± 0.0064		0.5086 ± 0.0103		1.4615 ± 0.0057		37.9998 ± 0.0034			1.6129 ± 0.0001	
Mannitol-P5	88.47	0.33 ± 0.03		0.3153 ± 0.0064		0.5682 ± 0.0145		1.4679 ± 0.0024		35.3318 ± 1.1549			1.5467 ± 0.0273	
**Materials**	**AR (°)**		**d (0.5) (μm)**		**Span**		**Uniformity**		**SA-BET (m^2^/g)**		**SA-BJH (m^2^/g)**	**PV (cm^3^/g)**		**PD (nm)**
Mannitol	45.37 ± 0.65		75.687 ± 1.067		1.332 ± 0.021		0.402 ± 0.008		0.3978 ± 0.0051		0.4496	0.000508		45.217
Mannitol-P0	53.12 ± 0.47		64.972 ± 3.561		1.947 ± 0.129		0.624 ± 0.043		0.6015 ± 0.0041		0.4955	0.001369		110.515
Mannitol-P0’	54.20 ± 0.46		44.099 ± 1.434		2.874 ± 0.288		0.879 ± 0.081		0.7792 ± 0.0086		0.5175	0.001583		107.384
Mannitol-P1	55.46 ± 0.42		43.069 ± 1.712		2.199 ± 0.231		0.683 ± 0.070		1.3201 ± 0.0115		0.9639	0.002918		121.089
Mannitol-P3	53.93 ± 0.46		51.138 ± 0.667		1.913 ± 0.118		0.592 ± 0.034		2.1634 ± 0.0127		1.7327	0.003822		88.244
Mannitol-P5	53.40 ± 0.47		46.229 ± 1.043		1.968 ± 0.037		0.622 ± 0.009		1.1867 ± 0.0171		0.5206	0.001359		119.365

Mannitol, the raw material; PVP, polyvinylpyrrolidone K30; Mannitol-P0, prepared without template agent PVP and ethanol; Mannitol-P0’, processed without PVP but with ethanol; Mannitol-P1, processed with 1% PVP; Mannitol-P3, processed with 3% PVP; Mannitol-P5, processed with 5% PVP. MC, moisture content; *ρ_b_*, bulk density; *ρ_t_*, tapped density; *ρ_true_*, true density; CI, Carr’s index; HR, Hausner ratio; AR, angle of repose; d (0.5), median particle size; span, particle size consistency; SA-BET, the surface area characterized by BET; SA-BJH, BJH adsorption cumulative surface area of pores between 17 nm and 3000 nm in diameter; PV, BJH adsorption cumulative volume of pores between 17 nm and 3000 nm in diameter; PD, BJH adsorption average pore diameter.

**Table 2:** The surface tension of the solution studied ($\bar{X}$ ± SD, *n* = 3).

Materials	Surface Tension (mN/m)
Mannitol-P0	69.967 ± 0.802
1% PVP	60.100 ± 0.854
3% PVP	56.867 ± 0.896
5% PVP	52.867 ± 0.569
Mannitol-P1	55.267 ± 1.168
Mannitol-P3	53.767 ± 0.451
Mannitol-P5	54.467 ± 1.050

PVP, polyvinylpyrrolidone K30; Mannitol-P0, prepared without template agent PVP and ethanol; Mannitol-P1, processed with 1% PVP; Mannitol-P3, processed with 3% PVP; Mannitol-P5, processed with 5% PVP.

**Table 3:** Fitting parameters of quasi-first order adsorption kinetics and quasi-second order adsorption kinetics of curcumin and ibuprofen.

		Curcumin		Ibuprofen	
Adsorption Kinetics Model	Samples	Regression Equation	R^2^	Regression Equation	R^2^
Quasi-first order	Mannitol	In (0.0046 − *Q_t_*) = −0.0039*t* − 5.3817	0.9093	In (1.5700 − *Q_t_*) = −0.0069*t* + 0.4511	0.9153
	Mannitol-P0	In (0.0136 − *Q_t_*) = −0.0060*t* − 4.2977	0.5256	In (2.1410 − *Q_t_*) = −0.0062*t* + 0.7613	0.3951
	Mannitol-P0’	In (0.0080 − *Q_t_*) = −0.0039*t* − 4.8283	0.4910	In (2.4250 − *Q_t_*) = −0.0077*t* + 0.8858	0.9986
	Mannitol-P1	In (0.0205 − *Q_t_*) = −0.0125*t* − 3.8873	0.7874	In (8.4220 − *Q_t_*) = −0.0070*t* + 2.1309	0.9419
	Mannitol-P3	In (0.0194 − *Q_t_*) = −0.0102*t* − 3.9425	0.9924	In (3.5690 − *Q_t_*) = −0.0083*t* + 1.2722	0.9476
	Mannitol-P5	In (0.0203 − *Q_t_*) = −0.0080*t* − 3.8971	0.9711	In (4.9970 − *Q_t_*) = −0.0057*t* + 1.6088	0.9068
Quasi-second order	Mannitol	*t*/*Q_t_* = 216.4700*t* + 8282.9000	0.9467	*t*/*Q_t_* = 0.6092*t* + 15.9730	0.9861
	Mannitol-P0	*t*/*Q_t_* = 76.0020*t* + 705.8300	0.9940	*t*/*Q_t_* = 0.4323*t* + 13.2390	0.9611
	Mannitol-P0’	*t*/*Q_t_* = 76.0020*t* + 705.8300	0.9873	*t*/*Q_t_* = 0.3665*t* + 16.5580	0.9727
	Mannitol-P1	*t*/*Q_t_* = 42.0340*t* + 2764.4000	0.9574	*t*/*Q_t_* = 0.1080*t* + 6.2784	0.9751
	Mannitol-P3	*t*/*Q_t_* = 43.1880*t* + 4627.8000	0.9438	*t*/*Q_t_* = 0.2245*t* + 28.5270	0.8865
	Mannitol-P5	*t*/*Q_t_* = 43.9050*t* + 3262.0000	0.9679	*t*/*Q_t_* = 0.1844*t* + 9.7422	0.9824

Mannitol, the raw material; PVP, polyvinylpyrrolidone K30; Mannitol-P0, prepared without template agent PVP and ethanol; Mannitol-P0’, processed without PVP but with ethanol; Mannitol-P1, processed with 1% PVP; Mannitol-P3, processed with 3% PVP; Mannitol-P5, processed with 5% PVP. *Q_t_* was expressed as the adsorption capacity of the sample at any time *t* (min), in mg/g; *t* was the adsorption reaction time (min).

**Table 4:** The maximum cumulative release rate of materials.

Materials	Release in 120 min (%)
Mannitol-Cur	28.39 ± 3.49 ^de^
Mannitol-P0-Cur	37.38 ± 5.13 ^c^
Mannitol-P0’-Cur	34.55 ± 6.18 ^cd^
Mannitol-P1-Cur	62.44 ± 1.47 ^b^
Mannitol-P3-Cur	69.44 ± 0.81 ^a^
Mannitol-P5-Cur	69.82 ± 0.96 ^a^
Mannitol-Ibu	27.65 ± 0.92 ^e^
Mannitol-P0-Ibu	28.10 ± 1.76 ^e^
Mannitol-P0’-Ibu	29.94 ± 2.53 ^de^
Mannitol-P1-Ibu	68.22 ± 3.28 ^ab^
Mannitol-P3-Ibu	71.58 ± 3.07 ^a^
Mannitol-P5-Ibu	65.37 ± 0.78 ^ab^

Data are expressed as mean ± standard deviation, and different lowercase letters represent significant differences (*p* < 0.05).

**Table 5:** Fitting parameters of drug release models determined with different mathematical models.

	Mathematical Models		
Materials	Zero-Order	First-Order	Korsmeyer–Peppas
Mannitol-Cur	*Q* = 0.2664*t* − 3.9342, R^2^ = 0.8594	100 − *Q* = 100exp^(−0.0024*t*)^, R^2^ = 0.8979	*Q* = 0.0777*t*^0.4276^, R^2^ = 0.9110
Mannitol-P0-Cur	*Q* = 0.2956*t* − 0.7457, R^2^ = 0.8396	100 − *Q* = 100exp^(−0.0034*t*)^, R^2^ = 0.7848	*Q* = 0.5762*t*^0.3436^, R^2^ = 0.8545
Mannitol-P0’-Cur	*Q* = 0.2888*t* − 0.0220, R^2^ = 0.8545	100 − *Q* = 100exp^(−0.0034*t*)^, R^2^ = 0.8033	*Q* = 0.6733*t*^0.4106^, R^2^ = 0.8809
Mannitol-P1-Cur	*Q* = 0.4894*t* + 23.9322, R^2^ = 0.5949	100 − *Q* = 100exp^(−0.0177*t*)^, R^2^ = 0.7737	*Q* = 10.1192*t*^0.4282^, R^2^ = 0.7967
Mannitol-P3-Cur	*Q* = 0.4973*t* + 24.0012, R^2^ = 0.6423	100 − *Q* = 100exp^(−0.0178*t*)^, R^2^ = 0.8016	*Q* = 10.0295*t*^0.4323^, R^2^ = 0.8273
Mannitol-P5-Cur	*Q* = 0.4949*t* + 17.5228, R^2^ = 0.7821	100 − *Q* = 100exp^(−0.0132*t*)^, R^2^ = 0.8773	*Q* = 6.6676*t*^0.4990^, R^2^ = 0.9074
Mannitol-Ibu	*Q* = 0.2718*t* − 0.9126, R^2^ = 0.9084	100 − *Q* = 100exp^(−0.0030*t*)^, R^2^ = 0.8332	*Q* = 0.4215*t*^0.4923^, R^2^ = 0.9126
Mannitol-P0-Ibu	*Q* = 0.2749*t* − 0.7369, R^2^ = 0.9172	100 − *Q* = 100exp^(−0.0031*t*)^, R^2^ = 0.8408	*Q* = 0.4192*t*^0.3980^, R^2^ = 0.9217
Mannitol-P0’-Ibu	*Q* = 0.3060*t* − 1.3295, R^2^ = 0.8954	100 − *Q* = 100exp^(−0.0034*t*)^, R^2^ = 0.8220	*Q* = 0.4570*t*^0.3977^, R^2^ = 0.8966
Mannitol-P1-Ibu	*Q* = 0.4878*t* + 23.2176, R^2^ = 0.6374	100 − *Q* = 100exp^(−0.0169*t*)^, R^2^ = 0.7952	*Q* = 9.7155*t*^0.4341^, R^2^ = 0.8312
Mannitol-P3-Ibu	*Q* = 0.4691*t* + 26.1433, R^2^ = 0.6080	100 − *Q* = 100exp^(−0.0177*t*)^, R^2^ = 0.7570	*Q* = 11.3715*t*^0.4027^, R^2^ = 0.8802
Mannitol-P5-Ibu	*Q* = 0.4841*t* + 17.8794, R^2^ = 0.8188	100 − *Q* = 100exp^(−0.0129*t*)^, R^2^ = 0.8793	*Q* = 6.8218*t*^0.4916^, R^2^ = 0.9365

*Q* represents the cumulative dissolution percentage of the sample, and *t* is the release time.

## Data Availability

The data presented in this study are available on request from the corresponding author.

## Supplementary Materials

The following supporting information can be downloaded at: https://www.mdpi.com/article/10.3390/molecules29030715/s1, Figure S1: SEM images of mannitol before and after drug loading (500×). Table S1: Overview of the primary sequence and molecular weight of each compound.
