# Supplementary material for: The Fabrication, Drug Loading, and Release Behavior of Porous Mannitol
Source: Molecules. 2024 Feb 4;29(3):715. doi: 10.3390/molecules29030715 (PMC10856056; doi:10.3390/molecules29030715)
Supplement: Supplementary file 1 [file molecules-29-00715-s001.zip › molecules-2826598-supplementary.pdf]

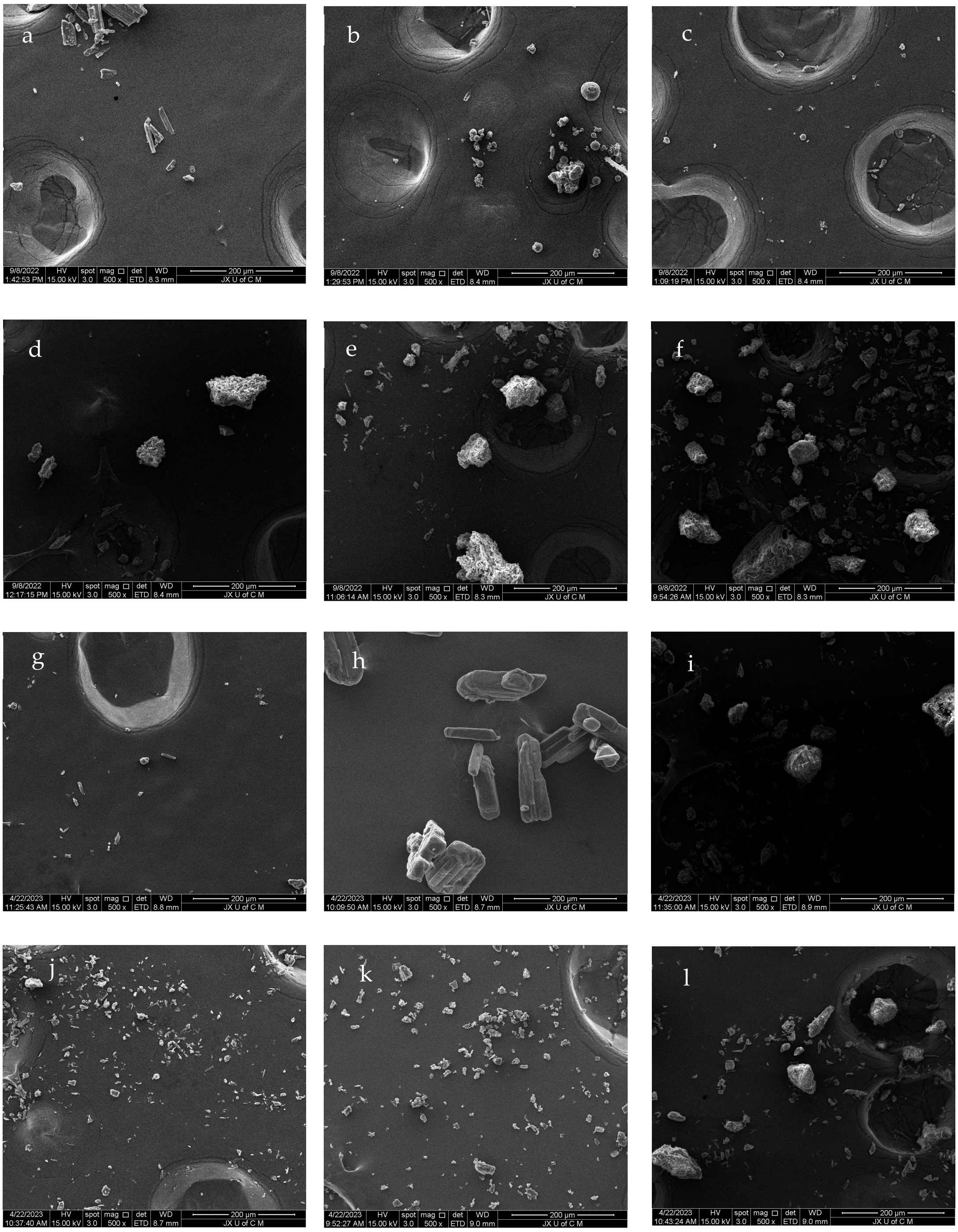

Figure S1. SEM images of mannitol before and after drug loading (500×). **(a)** Mannitol, the raw material; **(b)** Mannitol-P0, prepared without template agent PVP and ethanol; **(c)** Mannitol-P0', processed without PVP but with ethanol; **(d)** Mannitol-P1, processed with 1% PVP; **(e)** Mannitol-P3, processed with 3% PVP; **(f)** Mannitol-P5, processed with 5% PVP; **(g)** Mannitol-P0'-Cur, the Mannitol-P0' loaded with curcumin; **(h)** Cur, the model drug of curcumin; **(i)** Mannitol-P3-Cur, the Mannitol-P3 loaded with curcumin; **(j)** Mannitol-P0'-Ibu, the Mannitol-P0' loaded with ibuprofen; **(k)** Ibu, the model drug of ibuprofen; **(l)** Mannitol-P3-Ibu, the Mannitol-P3 loaded with ibuprofen.

Table S1. Overview of the primary sequence and molecular weight of each compound.

Compound	Sequence	Molecular weight (g/mol)
Mannitol	FBPFZTCFMRRESA−KVTDHHQDSA−N	182.17
PVP	/	111.14
Curcumin	VFLDPWHFBUODDF−FCXRPNKRSA−N	368.40
Ibuprofen	HEFNNWSXXWATRW−UHFFFAOYSA−N	206.28
KBr	IOLCXVTUBQKXJR−UHFFFAOYSA−M	119.00
